# Ecological Thresholds in the Savanna Landscape: Developing a Protocol for Monitoring the Change in Composition and Utilisation of Large Trees

**DOI:** 10.1371/journal.pone.0003979

**Published:** 2008-12-18

**Authors:** Dave J. Druce, Graeme Shannon, Bruce R. Page, Rina Grant, Rob Slotow

**Affiliations:** 1 Amarula Elephant Research Programme, Biological and Conservation Sciences, Westville Campus, University of KwaZulu-Natal, Durban, South Africa; 2 Scientific Services, Kruger National Park, Skukuza, South Africa; University of Bristol, United Kingdom

## Abstract

**Background:**

Acquiring greater understanding of the factors causing changes in vegetation structure - particularly with the potential to cause regime shifts - is important in adaptively managed conservation areas. Large trees (≥5 m in height) play an important ecosystem function, and are associated with a stable ecological state in the African savanna. There is concern that large tree densities are declining in a number of protected areas, including the Kruger National Park, South Africa. In this paper the results of a field study designed to monitor change in a savanna system are presented and discussed.

**Methodology/Principal Findings:**

Developing the first phase of a monitoring protocol to measure the change in tree species composition, density and size distribution, whilst also identifying factors driving change. A central issue is the discrete spatial distribution of large trees in the landscape, making point sampling approaches relatively ineffective. Accordingly, fourteen 10 m wide transects were aligned perpendicular to large rivers (3.0–6.6 km in length) and eight transects were located at fixed-point photographic locations (1.0–1.6 km in length). Using accumulation curves, we established that the majority of tree species were sampled within 3 km. Furthermore, the key ecological drivers (e.g. fire, herbivory, drought and disease) which influence large tree use and impact were also recorded within 3 km.

**Conclusions/Significance:**

The technique presented provides an effective method for monitoring changes in large tree abundance, size distribution and use by the main ecological drivers across the savanna landscape. However, the monitoring of rare tree species would require individual marking approaches due to their low densities and specific habitat requirements. Repeat sampling intervals would vary depending on the factor of concern and proposed management mitigation. Once a monitoring protocol has been identified and evaluated, the next stage is to integrate that protocol into a decision-making system, which highlights potential leading indicators of change. Frequent monitoring would be required to establish the rate and direction of change. This approach may be useful in generating monitoring protocols for other dynamic systems.

## Introduction

Historically, decisions in conservation management were based predominantly on the experience of wildlife managers. This trend has changed with environmental and conservation decision-making now being informed by science [1], [2], which, combined with democratic societal values, results in a need for transparency in both conservation planning and management. Accountability has led to the drafting of objective-driven park conservation plans (e.g. [3], [4], [1]). Furthermore, many parks subscribe to an active interventionist approach to perturb the system in a direction required to meet stated objectives [3], [5]. Any such approach, and particularly one that pursues active adaptive management, requires monitoring of key indicators in order to gauge progress (e.g. [6], [1]).

Of particular concern in conservation management is the potential for regime shifts, whereby ecosystems change from one state to another under pressure from one or more ecological drivers (see [7], [8]). These changes can be both rapid and irreversible, depending upon the system in question ([7]). Therefore, conservation managers need to understand where a particular system is in the stability landscape (i.e. how close is it to a change in state), and the resilience of that system (i.e. the amount of disturbance that can be tolerated without undergoing a regime shift see [9]). The success of managing complex ecological systems depends largely on the strength of the monitoring approaches and the implementation of adaptive strategies to mitigate potential regime shifts [2]. Ideally, leading indicators of impending regime shift are required to forewarn management of major changes in the ecosystem [8], [9]. The problem however is that ecological thresholds can be difficult to discern and suitable indicators, even generic ones (e.g. [8]), are poorly understood and remain largely a theoretical concept [10], [11] As a result, a monitoring approach needs to be established which balances the practicalities of short to medium term ecosystem management with the long-term data requirements needed to explore complex ecological theory [11].

The Kruger National Park (KNP), South Africa recently revised their management plan [4] and included a framework for decision-making based on Thresholds of Potential Concern (TPCs) and adaptive management [3]. TPCs are defined as those upper and lower levels along a continuum of change in a selected environmental indicator which, when reached, prompts an assessment of the causes which led to such an extent of change. The outcome of the TPC process results in either management action to moderate such causes, or re-calibration of the threshold to a more meaningful or realistic level [12]. TPCs have been developed in order to provide an early warning system with regard to unacceptable system changes or potential losses of biodiversity as a result of drivers, such as water provision, fire, herbivory and climate change [4]. TPCs are, therefore, pre-determined limits set for various components of the environment, with management mitigation implemented once these limits are reached. In many cases these limits can not be defined by an exact figure, but are defined once a trend develops and more data are available. In order to determine when these levels are being approached or exceeded, monitoring programs have to be developed which can reliably document environmental variation over time.

Savanna ecosystems such as the KNP are defined by a continuous herbaceous layer interspersed with trees, representing a system of regime shifts between adaptive states (reviewed in [7]). Large trees play a particularly important role in the savanna ecosystem, from the plant to landscape scale. They promote species diversity and provide an important component of spatial heterogeneity in habitat structure, while also acting as nutrient pumps creating islands of fertility within the savanna landscape [13]–[16]. The African elephant (*Loxodonta africana*) and fire have been identified as the two main drivers responsible for the mortality of large trees resulting in the modification of habitat structure and composition within the savanna biome [17]–[23]. It is therefore postulated that when the large tree component of a savanna system is substantially reduced, the nature of the system is changed, and that it then exists in a different state [18]. It is believed that these alternative states are represented by stable equilibrium separated by a defined threshold; however this has not been definitively proved. Furthermore, regime shifts in ecological systems are very difficult to define and detect, requiring detailed long term data sets [11]. The TPC approach therefore attempts to set limits of acceptable change at levels above the boundaries that define regime shifts. The method is therefore adaptive and TPCs can be recalibrated over time as the boundaries defining alternative states are more accurately established ([3]).

Due to the perceived vegetation impacts of elephant, the population in KNP was previously maintained at between 6900 and 8700 individuals (the predicted carrying capacity) through annual culling operations (1967–1994) [24], [12]. In 1995 culling was ceased due to increasing public pressure and a lack of definitive scientific evidence with regard to elephant driven impacts [12]. As a result, the elephant population grew to an estimated 13352 by 2007 [25]. This situation has lead to concern about the possible impacts that the increasing elephant population is having on habitat structure and biodiversity of KNP [26]. Previous monitoring had already indicated that large trees (≥5 m in height) were declining in abundance across large areas of KNP [27], and this has led to the inclusion of a structure specific TPC in the KNP management plan [1] which addresses both the abundance and diversity of large trees. A monitoring protocol is now required, which measures changes in the large tree component, as well as identifying ecological drivers of such change. This is particularly important in this instance, as elephants may not be the only ecological driver causing a decline in large trees [21]. Moreover, exceeding this TPC may trigger a management intervention to reduce elephant numbers, a very contentious issue [26], [1] Therefore a useful monitoring protocol also needs to be able to reliably distinguish the difference between natural variability in rates of change and rates induced by perturbations, which can drive the system towards a regime shift.

Previous studies on the vegetation use by elephant have often only been undertaken once, or focused on a single factor (e.g. [28], [29]–[31]). However, various approaches have been used to extrapolate the historical change in vegetation structure and composition (see [32]), including pollen and charcoal analysis [33] and long period repeat aerial photographs [27], [34]. Although some studies have been repeated annually for a number of years, they may not have re-sampled the same sites (e.g. [20]), while others were conducted too infrequently resulting in speculation as to the possible reasons for change (e.g. [22], [34]).

Sampling designs also raise problems, for example, Calenge et al. [28] used 32 m circular plots which limited their ability to test the full range of factors affecting spatial patterns of tree damage. Some studies have been confounded by biases introduced by roads used as corridors by elephant (e.g.[29], [30], [35]). Thus it has generally proven difficult to identify the possible factors causing changes in woody vegetation structure, composition and cover over time [27], [36].

To establish both the credibility and acceptability of monitoring programmes, peer-review publication of all phases is recommended [37]. In this paper we document the identification of indicators in the design phase of Reyers and McGeogh's [37] approach to the development of a monitoring protocol for this regionally important conservation issue [26], [1]. We undertook a study in the Southern Section of the KNP during April 2006, to develop an effective method for sampling changes in abundance and size distribution of large trees (≥5 m in height) and identification of drivers responsible for the changes. To achieve this the method had to: (1) effectively determine the density of individuals ≥5 m in height across different tree species and height classes present within a designated area, (2) identify all types of large herbivore use on the individual trees sampled, (3) determine changes in the state/growth of individual trees over time, (4) identify factors driving change in the abundance and size distribution of large trees, (5) be efficient in terms of manpower and sampling time, and (6) provide a framework for developing potential leading indicators of regime shift in this system. As a result, the aim of this study was to test a sampling strategy which would satisfy as many of these criteria as possible and to make recommendations about future sampling methods based on experience gained from this study. The ecological results of this study are presented elsewhere [21], [38].

## Results

**Figure 1:**
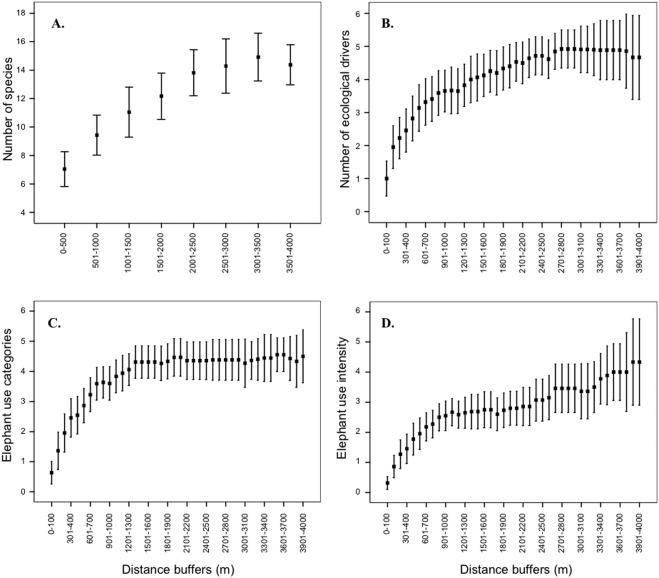
A measure of effectiveness for monitoring ecological impact on large trees. (a) Accumulation curve (mean±95% confidence limits) of tree species within increasing 500 m buffers to determine optimal distance (observed as the point at which the dependent variable levels off) to sample all tree species. Increasing 100 m buffers were used to produce accumulation curves to determine the optimal distance to sample all (b) ecological drivers, (c) elephant use categories and (d) elephant intensity of use categories.

The shortest transect was 900 m in length and the longest was 6.6 km. A maximum of 20 tree species were sampled within a transect (mean±SE: 14±1 tree species). On average, a transect of 3 km was required to include 14 tree species (Fig. 1a), while just under five of the six key ecological drivers (Table 1) could be detected within the first 3.0 km (Fig. 1b). However, almost all elephant use types present within any particular transect could be detected within the first 1.3 km (Fig. 1c). Although the number of elephant intensity of use categories that could be detected within the first 400 m was high, these seemed to reach a slight plateau by 2 km (just over three categories) and then only slowly increased thereafter to the maximum number of intensity of use categories of four, which were recorded within the 4 km buffer (Fig. 1d). Five trees exhibiting no impact were detected in the shortest length of transect, while five trees with primary branches removed by elephant took the longest length of transect to detect (Table 1). Trees (five individuals) most likely to be removed from the system as a result of elephant use (pushed over and/or those which had more than 50% of their bark removed) were detected within a mean distance of 1.7 km. Elephant use of at least five individuals was detected in the shortest distance (mean 260 m) along the transect, whilst the occurrence of disease was significantly rarer, requiring approximately 1.8 km of transect to locate five individuals (Table 1).

**Table 1:** The number of transects and the average distance required to sample five individual trees exhibiting the same type of elephant use, to sample five individuals used or modified by the same ecological driver, to sample five individuals exhibiting use less and greater than six months and to sample five individuals of three locally abundant tree species.

	Number of transects containing five or more individuals		Average distance (m) along transect to sample five individuals. Standard error in parentheses.
Utilised individuals	River transects (total of 14)^1^	Photo transects (total of 8)^2^	
Pushed over or broken and either dead or alive	10	8	923 (±135)
Main trunk tusk gashed or debarked	14	8	731 (±156)
Roots exposed and eaten	0	3	933 (±328)
Primary branches broken	14	4	1105 (±313)
Secondary and/or smaller branches broken	14	7	438 (±145)
High impact (main trunk debarked ≥50% and/or pushed over)	8	7	1720 (±313)
No obvious utilisation	14	8	318 (±56)
**Ecological drivers**			
Elephant	14	8	259 (±76)
Giraffe	13	8	538 (±98)
Other browsers	12	7	889 (±119)
Disease	13	4	1817 (±330)
Fire	7	6	746 (±259)
Natural dieback	13	7	520 (±138)
**Age of utilisation**			
Less than 6 months	12	4	1187 (±259)
Older than 6 months	14	8	190 (±49)
**Tree species**			
*Acacia nigrescens*	10	6	693 (±147)
*Combretum apiculatum*	10	5	1326 (±216)
*Sclerocarya birrea*	9	4	1076 (153)

1 Transects which ran perpendicular from watercourses.

2 Transects aligned with existing fixed-point photographs.

**Figure 2:**
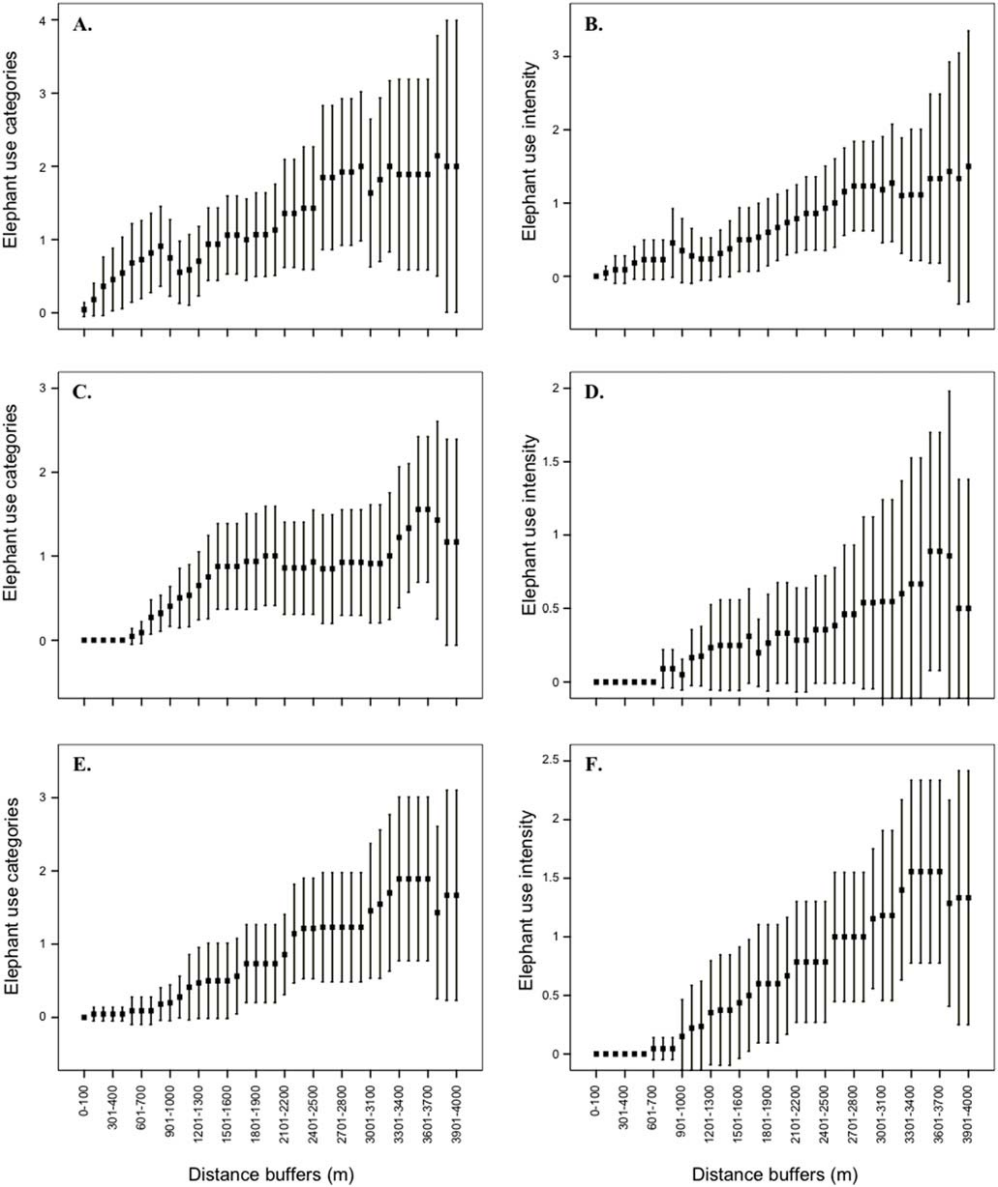
Accumulation curves (mean±95% confidence limits) within increasing 100 m buffers to determine optimal distance (observed as the point at which the dependent variable levels off) required to sample elephant (a) use categories and (b) intensity of use categories for *Acacia nigrescens*, (c) elephant use categories and (d) intensity of use categories for *Combretum apiculatum* and (e) elephant use categories and (f) intensity of use categories for *Sclerocarya birrea*. Note the greater variation for single species accumulation curves (compared with Fig. 1) and the increased distance required to approach an optimal transect length.

From an individual species perspective, five *Acacia nigrescens* trees were located within the shortest distance, and were also sampled in more transects than the other two focus tree species (Table 1). The greatest transect distance was required to locate five *Combretum apiculatum* individuals (Table 1). Interestingly, this may be due to the location of transects and the preference of *C. apiculatum* to slopes and crests. However, establishing these influences is beyond the scope of this study. Both the number of elephant use categories and the intensity of use categories continued to increase along the length of the transects for *A. nigrescens* (Fig. 2a and 2b), *C. apiculatum* (Fig. 2c and 2d) and for *Sclerocarya birrea* (Fig 2e and 2f). The individual height classes need assessed across the entire data set due to the predominance of trees in the small height class (92% are <10 m in height. See [21]).

With regard to manpower, an average of 3.06 (±0.2, N = 19 transects) man-h was required to sample 1 km of large trees (≥5 m). In essence, this method requires three people and associated logistics (vehicle etc.) for a full day to conduct one transect of about 3 km. This includes breaks and the time taken to return to the vehicle. Data-capture and summary analysis took approximately 3 h per 3 km transect.

## Discussion

Over the past 40 years a number of studies have focussed solely on the effects of elephants on vegetation [28], [35], [39]–[45] and biodiversity [46] (for review see [47]), as a result of what has been referred to as “the elephant problem”. More recently an integrated approach has been pursued, for example determining the interaction of elephants and fire [23], [48], [49] and the influence of elephants, drought and the provision of artificial water sources [50] on savanna vegetation structure. The method assessed in this study is compatible with such an integrated approach as it aims to distinguish the role of key ecological drivers in the use of large trees over time.

Previously, sampling approaches aimed at determining the factors driving vegetation change have often been inconsistent, and directed more by short-term scientific objectives rather than long-term conservation objectives. Effective monitoring requires consistency and relevance to objectives, whilst ensuring compatibility. All too frequently, fragmented sampling approaches prevent meta-analysis across conservation units, which is essential for understanding broader drivers such as climatic effects. Furthermore, in order to establish the correct monitoring programmes an initial phase in which the relevant objective(s) are set is required [37]. This has been successfully carried out in the KNP, where a threat to large trees has been identified as jeopardising conservation objectives [1].

Monitoring approaches need to take into account the spatial and temporal heterogeneity of the system in order to prevent a mismatch of scale between ecological process and management intervention [51]. KNP monitoring units are set at the landscape scale [3] and our tested method is one that samples broadly and continuously across habitats (see [38]). Relatively large distances need to be covered in order to sample the variety of tree species present, their density, and the different size classes, particularly considering their discrete distribution. It is important to emphasize that the comparatively long transects tested in this study also enhances the ability to identify agents of mortality and the ultimate causes of change. For example, repeat sampling of long transects over varied relief will enable researchers to distinguish between mortality from moisture limitation and senescence or frost. This approach requires taking cognisance of the relief at the point at which an individual occurs (i.e. hilltops are usually drier than valley bottoms), coupled with data on recent climatic events (e.g. drought). Furthermore elephant use is predicted to exhibit pronounced heterogeneity at a range of scales (landscape, habitat and patch [38]) and therefore a sampling regime needs to be able to account for this variation.

The accumulation curves provided a very effective method for assessing the optimum length of the large tree transects. Ideally, all species and height classes would be sampled along with the use/impact of each ecological driver. However the sampling effort required would be prohibitive, particularly with regard to the large height classes (>10 m in height), which are comparatively rare. Therefore an optimal approach is required, which provides effective sampling coverage in the minimum distance. The diminishing rates of return after 3 km for the large tree community (numbers of species and ecological drivers sampled) suggests that this is a suitable and ecologically meaningful transect length. Interestingly, elephant use categories have been well sampled by 1.6 km, however this does not allow for an integrated approach with regard to the sampling of other ecological drivers (e.g. fire, disease and other herbivores). In conclusion therefore a 3 km transect is large enough to reliably sample large tree abundance and use across the broad landscape types of KNP whilst also being an achievable daily objective for two trained field workers. In a three-week period the two researchers collecting the data for this study were able to complete 67 km of transects, which included 3082 individual large trees, providing a detailed level of information on the current status of large trees across the habitats of the Southern KNP [21].

It is essential that the objectives and overall aim of the monitoring protocol be expressed explicitly so that sufficient data are collected. For example, whilst the 3 km sampling approach is effective for assessing the distribution, abundance and use of the large tree community as a whole, it is not long enough to provide the same detail of information on individual tree species (*A. nigrescens*, *C. apiculatum* and *S. birrea*). There is also a much greater level of variation associated with analysing each species separately due to their unique habitat and environmental preferences. Greater elucidation may well be achieved from considering the entire dataset, which is also recommended for the individual height classes. Moreover the long transect approach is not suited to sampling rare tree species due to their discrete distribution and low abundance. Therefore an individual species based approach with repeat sampling of known individuals is required to determine their dynamics and use [21].

In a number of situations, ecological drivers work in tandem to cause the eventual mortality of an individual tree [18]. For example, elephants may debark a section of the tree, allowing wood boring invertebrates to enter, opening up a section of the stem, and making it more susceptible to fire. Consequently, fire may be the eventual cause of death, although the process began with elephants stripping the bark a few years previously. In cases of multiple causation only shorter interval re-sampling of specific, marked, individuals will resolve the sequence of agents and/or processes causing mortality and thus ultimately driving change (see [52]). A minimum interval of 12 months is reasonable for such sampling, and given seasonal differences, probably the most sensible interval for repeat, intensive, sampling of marked individuals.

The decline of large trees has previously been documented in KNP [27] but the ecological causation and implications of this decline are not fully understood. For example, it has been suggested that large trees have become dominant in the savanna landscape over the past 100 years as a result of significant reduction in large herbivore populations due to excessive hunting and disease (e.g. rinderpest) [53]. Therefore, the observed change in abundance may be due to the equilibrium re-establishing itself as populations of large herbivores increase rather than as a result of system collapse or a change in state [54]. Nonetheless, the large tree component of the savanna plays a fundamental role in ecosystem function and effective, repeat monitoring is required to inform conservation managers of changes in abundance and size distribution over time. This is particularly pertinent as conservation approaches need to be both accountable and precautionary. However, in order to establish an ecologically valid TPC it is important to determine the rate of change and the potential implications of change for biodiversity and ecosystem function. It is therefore essential that the monitoring protocol should not only be able to detect change, but also provide information for the recalibration of the TPC as knowledge of the system improves over time.

Meanwhile from a theoretical perspective, achieving greater understanding of ecological thresholds and the potential for regime shifts in the savanna ecosystem requires accurate data that track change in a leading indicator over time and at different spatial scales. At present, this study only provides a snap-shot of large tree abundance and distribution, and on its own can not be used to determine the resilience or stability of the savanna habitat in Southern KNP. In fact the complexity of the savanna biome is such that establishing leading indicators of regime shift may well require data to be collected for a number of decades before the system and the stability landscape is properly understood [11]. Furthermore, the suitability of large trees as leading indicators still needs to be established, particularly considering that they are long-lived and slow growing. Recent studies have suggested that ecological systems will begin to demonstrate increased variation and asymmetry close to an ecological threshold, and a suitable indicator needs to detect this prior to regime shift (see [10], [11]). This situation further highlights the challenge of not only managing ecosystems in the short-term to meet conservation objectives (implementation of adaptive TPCs) but also to fully understand the complexity of the system and the implications of change (ecological thresholds and regime shifts).

Several key issues have been highlighted within this study: Firstly, monitoring is an essential part of adaptive management and a major effort is required to detect the long-term patterns of change in large tree structure. Secondly, the validity, appropriateness, effectiveness and efficiency of monitoring methods need to be evaluated to provide confidence and accountability in conservation management [2]. Such critical assessment and validation is rarely conducted, and should be subjected to peer-review process [37]. Thirdly, we have only completed part of the process for establishing monitoring protocols and it is important that alternative approaches be considered [37], particularly with regard to rare species and distinct habitat types (e.g. river lines). Once a monitoring approach is established the next step is to use these data to create a predictive TPC based on trajectories of change (*sensu* example in [1]). Based on our data, we need to ensure that some aspect of measuring large trees ultimately has the potential to be a leading indicator of a regime shift [8]. Once this is established it can then be linked to an explicit TPC based conservation decision-making process. An approach such as documented here may prove useful in generating monitoring protocols in other terrestrial systems.

## Materials and Methods

### Study area

The KNP is situated in the North East of South Africa adjacent to the Mozambique boundary. It is 350 km long (North to South) and 60 km wide (West to East) with a total extent of approximately 20 000 km^2^. Our study focussed on an area of approximately 2100 km^2^ in the Southern part of the park (25°28′–24°91′S; 31°95′–31°32′E). Geologically, KNP can roughly be divided in half longitudinally with basalts that give rise to clay soils of higher nutrient status in the East and granitic formations that give rise to sandier less fertile soils in the West [27], [55], [56]. The Southern section of KNP receives a mean annual rainfall of 750 mm [24] and experiences hot, wet summers and cooler, dry winters. The temperatures range from a mean minimum of 6°C in winter to a mean maximum of 33°C in summer [55].

KNP falls within the savanna biome, and consists of a variety of Lowveld Bushveld habitat types [57]. The study was confined to the southern section of the KNP which is classified into eight broad vegetation types *Acacia* thickets, riverline thickets, lowveld sour bushveld, mountain bushveld, mixed *Combretum/Terminalia* woodland, *Combretum* woodland, *Acacia* marula woodland and thornveld. Five perennial rivers flow West to East including the Sabie and the Crocodile Rivers in the Southern section. There are also a substantial number of ephemeral rivers that flow during the wet summer season in addition to point water sources such as springs, pans and artificially pumped water points [58].

### Data collection

The study was undertaken during April 2006 and involved sampling trees ≥5 m using two types of transects that were sampled on foot. The first type were transects that ran perpendicular to large watercourses (river transects). Fourteen of these transects were sampled. Each transect was 10 m wide, a minimum of 3 km long and was completed in one day. This allowed us to cover a large area that could contain a range of habitat types and environmental conditions such as the top of slopes and drainage lines. Transects were lengthened if a minimum of 150 trees had not been sampled within the 3 km transect. The length of the longest transect was 6.6 km and was the only transect to be completed over two days. A GPS (Garmin 12XL without differential correction) was used to plot the transects and the position of each tree sampled. Because of the difficulty of maintaining straight transects, all but the first transect were run along a fixed line of longitude or latitude. Although this restricted transect directions to North, South, East or West, it was easier to maintain a straight line through the bush. It also allowed us to use the GPS to establish the width of the transect, with one increment on the GPS equivalent to 1 m. This was confirmed by actual measurement of the width at regular intervals to maintain consistency. The second type of transects were shorter and aligned North, South, East or West away from the access road at existing fixed-point photograph sites. These transects were also 10 m wide and sampled in the same way as the river transects. This approach provided a method for comparing time series photographic data with actual field observations of tree abundance and use. Three to four photo transects could be completed in one day by two researchers.

The height of each tree was determined using one of the researchers as a 1.8 m scale below the tree, whilst the other stood at least 10–15 m away and estimated the height of the tree using a ruler to measure the relative height of the researcher and the tree. The same technique was used for measuring the height below canopy and the canopy diameter. Although the height measurements were estimates, they did not need to be more accurate, as trees were placed into 1.5 m height classes in all subsequent analyses. The stem diameter was measured using a tape measure 1 m above the ground and the number of stems was recorded. Use of the tree by elephant, giraffe and other browsers, and the impact of fire and disease were recorded along with the occurrence of natural die-back. The cause of die-back was not always identified and could have been a result of senescence, moisture stress or frost. All of these factors are hereafter referred to as use. Use of the tree was broken down into distinct categories depending on the parts of the tree targeted (small/secondary and large woody/primary branches, stem, bark, roots and the whole tree) and further classified according to the percentage of available biomass (canopy volume/bark/roots) that was removed. This was estimated for each category and subsequently separated into six broad classes (1–10%, 11–25%, 26–50%, 51–75%, 76–90% and 90–100%). The process was repeated for each of the ecological drivers that had used/impacted a particular tree. It was important to accurately assess each type of use, particularly as the mortality of large trees can be the result of an interaction between ecological drivers (e.g. fire and elephant). The age of use was also estimated as being either greater or less than six months old; this was done by comparing observed use with events (e.g. fire and elephant foraging) that had been accurately dated. In order to establish the resilience and regeneration of particular species and height classes, trees which were pushed over, snapped or burnt were further categorised on whether they were alive and re-sprouting or dead.

### Data analysis

A range of accumulation curves were produced, using SPSS 13, to determine the optimal distances needed to be covered by a transect in order to obtain five individual trees exhibiting the same use (biomass removed by a specific ecological driver) type or intensity of use. Five individuals were chosen throughout the analyses, as this provided a representative sample of large tree distribution and use whilst avoiding the confounding effects of using only one or two individuals. Distances were determined as either 100 m or 500 m buffers from the start of each transect. Accumulation curves were cut off at a distance of 4 km, as only two transects exceeded this length. Although ten categories of use were recorded during the field data collection, a number were combined during analysis to give a total of six broad categories: (1) pushed over or broken and either dead or alive, (2) main trunk tusk gashed or debarked, (3) roots exposed and eaten, (4) primary branches broken, (5) secondary and/or smaller branches broken and (6) no obvious use. The six intensity of use categories were also analysed.

The species accumulation curve was constructed purely from the total number of different tree species within each 500 m buffer. A longer buffer was chosen for this analysis in order to include a representative number of tree species. The three tree species most commonly targeted by elephants *Acacia nigrescens*, *Combretum apiculatum* and *Sclerocarya birrea* were examined in further detail [21]. These tree species, along with *Spirostachys africana* and *Terminalia sericea*, accounted for 65% of all the trees sampled [21].

We calculated the number of man-hours required to sample trees taller than 5 m by using the distance of each transect and the time, recorded by the GPS, from the beginning of the transect to the end of the transect. This was the actual time taken by the two researchers to complete the transect and excluded travelling time to and from the transect, breaks and the time taken to walk back to the vehicle once the transect had been completed.
